# Psychological Distress and Well-Being among Students of Health Disciplines: The Importance of Academic Satisfaction

**DOI:** 10.3390/ijerph18042151

**Published:** 2021-02-23

**Authors:** Jessica Franzen, Françoise Jermann, Paolo Ghisletta, Serge Rudaz, Guido Bondolfi, Nguyen Toan Tran

**Affiliations:** 1 School of Health Sciences Geneva, HES-SO University of Applied Sciences and Arts Western Switzerland, Avenue de Champel 47, 1206 Geneva, Switzerland; Jessica.Franzen@hotmail.fr; 2 Department of Psychiatry, Geneva University Hospitals, Boulevard de la Cluse 51, 1205 Geneva, Switzerland; Francoise.Jermann@hcuge.ch; 3 Faculty of Psychology, University of Geneva, Boulevard du Pont-d’Arve 40, 1211 Geneva, Switzerland; Paolo.Ghisletta@unige.ch; 4 Faculty of Psychology, Swiss Distance University Institute, 3900 Brig, Switzerland; 5 Swiss National Centre of Competence in Research LIVES, University of Geneva, 1211 Geneva, Switzerland; 6 School of Pharmaceutical Sciences, University of Geneva, Rue Michel-Servet 1, 1206 Geneva, Switzerland; Serge.Rudaz@unige.ch; 7 Faculty of Medicine, University of Geneva, Rue Michel-Servet 1, 1206 Geneva, Switzerland; nguyen-toan.tran@unige.ch; 8 Australian Centre for Public and Population Health Research, Faculty of Health, University of Technology Sydney, P.O. Box 123, Sydney, NSW 2007, Australia

**Keywords:** mental health, psychological well-being, depression, anxiety, stress, undergraduate students, bachelor’s degree students, student academic satisfaction

## Abstract

Background: Research on the mental health of students in health disciplines mainly focuses on psychological distress and nursing and medical students. This study aimed to investigate the psychological well-being and distress and related factors among undergraduate students training in eight different health-related tracks in Geneva, Switzerland. Methods: This cross-sectional study used established self-filled scales for anxiety, depression, stress, psychological well-being, and study satisfaction. Descriptive statistics and hierarchical regression analyses were applied. Results: In October 2019, out of 2835 invited students, 915 (32%) completed the survey. Lower academic satisfaction scores were strongly associated with depression (β = −0.26, *p* < 0.001), anxiety (β = −0.27, *p* < 0.001), and stress (β = −0.70, *p* < 0.001), while higher scores were associated with psychological well-being (β = 0.70, *p* < 0.001). Being female was strongly associated with anxiety and stress but not with depression or psychological well-being. Increased age was associated with enhanced psychological well-being. The nature of the academic training had a lesser impact on mental health and the academic year had none. Conclusion: Academic satisfaction strongly predicts depression, anxiety, stress, and psychological well-being. Training institutions should address the underlying factors that can improve students’ satisfaction with their studies while ensuring that they have access to psychosocial services that help them cope with mental distress and enhance their psychological well-being.

## 1. Introduction

The World Health Organization defines mental health as “a state of well-being in which an individual realizes his or her own abilities, can cope with the normal stresses of life, can work productively and is able to make a contribution to his or her community” [1]. There are, hence, two distinct dimensions in mental health: a positive dimension, corresponding to psychological well-being, and a negative dimension, including psychological distress and mental disorders.

Therefore, the evaluation of student mental health should investigate both dimensions by taking into account students’ psychological distress as well as their psychological well-being. However, most studies on student mental health have examined psychological distress only, typically assessed in terms of depression, anxiety, and stress. In general, university students report high psychological distress scores [2]. A study investigating the links between psychological distress and health behaviors found that depressive symptoms correlated in men with skipping breakfast and low sleep quality and in women with skipping breakfast, inadequate physical activity, and short or long sleep hours [3].

In health sciences training, much of the relevant research has focused on medical and nursing students. Several studies showed that nursing students report very high anxiety, stress, and depression scores [4,5,6]. Nursing students report more stress, anxiety, and depression than students from other disciplines and people in the labor force [4]. A qualitative study identified four sources of stress, including clinical practice, theoretical training, personal life, and social life [7], while other studies highlighted clinical practice as the primary stressor [4,8,9,10]. A systematic review of the literature found a very high depression and anxiety prevalence among medical students and a higher psychological distress level than in the general population [11].

Compared to research on students’ psychological distress, only a few studies examined their psychological well-being. One qualitative study found that most students had a good quality of life and were satisfied with their health and how they lived [4]. Another study found a relationship between psychological well-being and physical activity among nursing students [12].

Most of the research investigating contributing factors has examined risk factors for increased psychological distress. Gender stands out as a significant factor influencing anxiety, depression, and stress. In general, female students report higher anxiety and stress levels than their male counterparts [2]. The same is valid for female students in health disciplines with respect to general psychological distress [6,11]. To our knowledge, only one study found no gender differences [5]. The academic year could also be a determining factor, with first-year and second-year students being more stressed, depressed, and anxious than others (because of, among others, greater student dropout rates) [2,7,9,13,14,15], and with fourth-year students having lower depression scores than second- and third-year students [4].

Compared to the many studies on risk factors, only a few investigated protective factors. One study examined internal and external factors predicting psychological well-being in nursing students [16]. It showed that self-efficacy, resilience, mindfulness skills, and social support positively affected their psychological well-being. Another study showed that student academic satisfaction also played a significant role, with satisfied students reporting less stress, anxiety, and depression than dissatisfied students [2]. The association between stress and student satisfaction in college was also reported in an East Asian study [17].

In summary, much research on student mental health has focused on psychological distress, and only a few studies investigated psychological well-being. However, a holistic approach should examine both positive and negative dimensions, thereby including protective and risk factors. Additionally, studies have mostly focused on medical and nursing students. According to our understanding, no study considered students of other health disciplines, such as midwifery, physiotherapy, nutrition and dietetics, medical radiology technology, psychology, or pharmaceutical sciences, all highly related to health disciplines.

Therefore, the main objective of this research was to study the mental health status of bachelor’s degree students training in different health disciplines in Geneva, Switzerland, by exploring both psychological distress and well-being and related risk and protective factors.

## 2. Materials and Methods

### 2.1. Study Population and Setting

This was a cross-sectional study involving bachelor-level students enrolled in the 2019–2020 academic year at the Geneva School of Health Sciences and the Faculty of Medicine, School of Pharmaceutical Sciences, and Psychology Department of the University of Geneva. We applied random sampling stratified by health disciplines by inviting all the students to participate in the study in October 2019. There were no exclusion criteria.

### 2.2. Measurements

Socio-demographic data included age, gender, current academic year, and health discipline. The study used the following scales for perceived stress, anxiety, depression, psychological well-being, and satisfaction with studies.

### 2.3. Depression and Anxiety

The Hospital Anxiety and Depression Scale (HADS) was used to identify the presence of depression and anxiety symptoms and assess their severity [18,19]. The questionnaire consists of a depression subscale and an anxiety subscale, each with seven items that are rated from 0 to 3.

### 2.4. Perceived Stress

The 14-item Cohen Perceived Stress Scale (PSS) was used to assess students’ perceived stress or, put differently, the extent to which they generally perceive life situations as threatening [20]. Participants rate statements on a scale of 0 (never) to 4 (very often).

### 2.5. Psychological Well-Being

The Psychological Well-Being Scale (BEP) was used to assess participants’ psychological well-being [21,22]. This 18-item scale contains six dimensions: autonomy, environmental mastery, personal growth, positive relationships with others, purpose in life, and self-acceptance. Participants rate statements on a scale of 1 (disagree) to 6 (agree).

### 2.6. Academic Satisfaction

The Scale of Satisfaction with Studies (SSS) was used to measure student academic satisfaction [23]. This five-item scale measures an overall and subjective assessment of students’ quality of life in their educational setting. Participants rate statements on a scale of 1 (strongly disagree) to 7 (strongly agree).

### 2.7. Data Collection

Participant recruitment proceeded via e-mails sent by the student secretariats to complete lists of students. Interested students could participate in the study by logging onto a secure electronic site (EvaSys Education Survey Automation Suite version 7.1, Electric Paper Evaluation Systems GmbH, Lüneburg, Germany). After providing their informed consent, participants anonymously answered socio-demographic questions and the HADS, PSS, BEP, and SSS questionnaires. Data were collected shortly after the beginning of the academic year in October 2019. All data were handled confidentially and securely on EvaSys and archived on a hard drive located in a locked office only accessible to the investigators.

### 2.8. Statistical Analysis

Descriptive analyses were performed for demographic data, which were reported as means ± standard deviation (SD). Hierarchical linear regression analyses estimated the contribution of these potential predictors and were performed separately for each of the depression, anxiety, stress, and psychological well-being scales by entering four separate blocks of independent variables. The sequential entry of predictors was drawn from the findings of previous research and included gender and age (block 1) [2,6,11], to which we added the academic year of training (block 2) [2,4,7,9], the health discipline (block 3) [4], and academic satisfaction (block 4) [2]. Predictors were considered significant when their *p*-value was 0.05 or less. We evaluated the increase in *R*^2^ to determine significance between two consecutive blocks. There were no missing data as the electronic survey required mandatory answers to all the questions. All analyses were computed using SPSS version 25 (IBM, Armonk, NY, USA).

### 2.9. Ethics Statement

The Ethics Research Committee of the Geneva University Hospitals reviewed the study protocol and decided to waive the need for an internal review board review as the study involved students and was anonymous (reference number: 2019-00696).

## 3. Results

Out of the 2835 students invited to participate in the study, 915 (32%) completed the survey (an additional three were excluded from the analysis as they entered invalid birth years). A vast majority were women (*n* = 753, 82%)—the proportion of women in the overall sampling pool ranged from 64% in the Faculty of Medicine to 81% in the Psychology Department. Participants’ age ranged from 16 to 61 years, with a mean age of 22—the mean age of the overall sampling pool ranged from 21 (Faculty of Medicine and School of Pharmaceutical Sciences) to 24 years (School of Health Sciences Geneva). In addition to students of psychology, pharmaceutical sciences, and medicine, participants included students of the other health disciplines taught at the School of Health Sciences Geneva, including midwifery, nursing, physiotherapy, nutrition and dietetics, and medical radiology technology. However, we considered all School of Health Sciences students together because a finer analysis by discipline would have led to small subsample sizes. Gender, age, and scores of the depression/HADS, anxiety/HADS, stress/PSS, psychological well-being/BEP, and academic satisfaction/SSS scales are presented in Table 1.

Table 2 reports the linear hierarchical regressions results. For all outcomes, the first three blocks predicted minimal variance (from <1% to 4%, mean amount < 2%). Academic satisfaction was by far the strongest predictor, with *R*^2^ increases ranging from 16% to 26%. Lower academic satisfaction/SSS scores were strongly associated with depression (β = −0.26, *p* < 0.001), anxiety (β = −0.27, *p* < 0.001), and stress (β = −0.70, *p* < 0.001), while higher scores with psychological well-being (β = 0.70, *p* < 0.001). Female gender was also strongly associated with anxiety (β = −2.06, *p* < 0.001) and stress (β = −3.35, *p* < 0.001) but not with depression or psychological well-being. Increased age was associated with enhanced psychological well-being only (β = 0.22, *p* < 0.01). There were no marked differences between the different health disciplines in relation to depression and anxiety scores. However, psychology students reported higher stress (β = 1.98, *p* < 0.01) and lower psychological well-being (β = −2.75, *p* < 0.01) compared to participants from other disciplines. Participants from the School of Pharmaceutical Sciences reported lower scores of psychological well-being (β = −2.49, *p* < 0.05) than those from the Faculty of Medicine or School of Health Sciences Geneva. The academic years across the different bachelor’s degrees did not predict depression, anxiety, stress, and psychological well-being scores.

To check the robustness of our results, we re-ran the regression models after the exclusion of all first-year students. This reduced the sample size from 915 to 526 second- and third-year students. The new regression results were virtually identical to the original, with negligible effects of the first three blocks of predictors on all four dependent variables (*R*^2^ ranging from 0% to 6.9%, mean amount = 2.61%). Academic satisfaction was, again, by far the strongest predictor, with *R*^2^ increases ranging from 17% to 27%. The respective regression coefficients for academic satisfaction/SSS scores were −0.25 for depression, −0.29 for anxiety, −0.74 for stress, and 0.70 for psychological well-being (all *p* < 0.001), thus being almost identical to those of the entire sample.

## 4. Discussion

We sought to investigate the mental health status of bachelor’s degree students of different health disciplines and related risk and protective factors. First and foremost, satisfaction with studies was found to have a substantial bearing on depression, anxiety, stress, and psychological well-being. The role of gender was also significant, as women reported more anxiety and stress than men, while increased age, even within our young sample, was associated with greater psychological well-being. The nature of the academic track had a minor impact on student mental health. However, involvement in pharmaceutical sciences and psychology was associated with decreased psychological well-being and studying psychology with more stress. The academic year had no influence.

To our knowledge, this is the first study on this topic involving western European students. Other relevant studies originated from Australia, New Zealand, the United States, Canada, Turkey, Spain, Cyprus, and Pakistan. Our study also explored psychological well-being. Our study contributes to the growing body of evidence on the intersection between mental health and students’ academic satisfaction. Previous research includes studies conducted in South Korea, correlating satisfaction in college with stress [17], and in Turkey, which showed that students satisfied with their education had lower depression, anxiety, and stress scores than those who were not satisfied [2].

In general, our research findings aligned with those of previous studies in terms of gendered anxiety and stress levels and positive association between academic satisfaction and mental health [2,6,11,17]. In contrast, the study did not find first- or second-year students to be more depressed, anxious, and stressed than their more advanced counterparts [2,7,9,13,14,15]. Nor did the results show that nursing students and other students attending the School of Health Sciences Geneva were at increased risk for poorer mental health than their counterparts from psychology, medicine, or pharmaceutical sciences [4,5,6]. However, increased age was positively correlated with psychological well-being, which contrasts with psychological well-being research. For example, age was not a contributing factor in a study involving American university students in the USA [24]. In this specific case, a possible explanation could be that our study sample was comparatively older and had a broader age range of 45 years (mean age, SD, range: 22.2 ± 4.3 (16–61) vs. 19.8 ± 1.3 (17–29)) [24].

### 4.1. Student Academic Satisfaction

Student satisfaction has been conceptualized as a favorable cognitive state influenced by a student’s positive educational experience according to Athiyaman, who drew the concept from consumer satisfaction models [25]. Understanding and meeting student expectations to improve their academic life satisfaction is challenging, as students bring multiple expectations to university. However, such iterative efforts are necessary, not least because of the potential impact of academic satisfaction on student psychological health and well-being.

Research has identified various personal, social, and structural factors associated with academic satisfaction and dissatisfaction. Exploring factors predicting undergraduate students’ satisfaction in the United Kingdom, Neville and Rhodes identified eight facets perceived to be deeply satisfying. They comprised the balance between study and personal life, the availability of learning resources, society’s views of students, feeling able to cope with the workload, the physical condition of the learning environment, feeling able to receive financial advice, the variety of assessment techniques, and other students’ views of university life [26]. The reputation of the program, quality of teaching, student-to-faculty ratios, faculty credentials, and student’s grades and performance were also found to influence academic satisfaction directly [27,28,29]. Other research underscored the significance of neuroticism on negatively predicting satisfaction with studies [30,31].

### 4.2. Implications for Policy, Practice, and Research

Owing to the importance of student academic satisfaction per se and as a predictor of psychological health and well-being in bachelor’s degree students of health disciplines, academic institutions should implement and evaluate specific interventions that address the factors mentioned above that influence student satisfaction. However, the needs of students experiencing psychological distress cannot be subsumed under satisfaction-enhancing interventions. They must be directly attended to by ensuring prompt access to quality psychosocial services whenever necessary. Tackling structural determinants of academic satisfaction, such as the physical learning environment, could be an early and more attainable gain than addressing students’ social and personal factors, including coping mechanisms or neuroticism.

Nonetheless, and by way of example, there is growing recognition that mindfulness-based interventions could play a determining role in addressing some of these factors among university students [32]. A randomized controlled trial showed that mindfulness training could improve problem-focused coping among Norwegian psychology and medical students [33]. The intervention also benefited those with higher neuroticism scores by reducing their avoidance-focused coping and increasing social support-seeking behaviors. Similarly, another Norwegian study demonstrated that mindfulness training could decrease neuroticism of medical and psychology students and, remarkably, over an extended follow-up period of six years [34]. The direct impact of mindfulness-based practice on students’ mental health has been demonstrated, such as in Spain, where mindfulness exercises were shown to help reduce stress and anxiety caused by exams in Bachelor of Education students [35]. Such mindfulness programs could be effective in decreasing stress, anxiety, and depression, even when delivered using an Internet platform, as reported, for instance, by a US study involving college students [36]. However, further research is needed to explore, specifically, the associations and causality links between various interventions, including mindfulness-based training, and student academic satisfaction.

### 4.3. Strengths and Limitations

Our study had several limitations. First, the survey occurred shortly after the beginning of the academic year, reflecting the stress and anxiety related to new beginnings, especially for first-year students. Surveying students at the end of a semester or academic year, when exams and deadlines often occur, may have yielded different results. Second, the self-administered survey offered only subjective measures. Third, the addition of a control group (students of similar demographic characteristics but non-health-related disciplines) would have allowed us to broaden the scope of the current investigation by supporting additional comparative conclusions. However, our main scope here was to study factors beyond psychological distress in multiple health-related disciplines (and not merely nursing and medical students). Fourth, the cross-sectional design could not exclude reverse causality in that lower psychological distress resulted in greater academic satisfaction. Fifth, it would have been interesting to assess whether students’ religiosity may have influenced our results given the effects of religiosity on life satisfaction [37].

There were several strengths to the study. First, it surveyed students of health disciplines other than medicine, nursing, or psychology. Second, it looked at both psychological distress and well-being. Third, it applied a rigorous statistical analysis approach using hierarchical regressions.

## 5. Conclusions

Academic satisfaction strongly predicts depression, anxiety, stress, and psychological well-being among bachelor’s students of health disciplines. Training institutions should address the underlying factors that can enhance students’ satisfaction with their studies in addition to ensuring that they have timely access to relevant psychosocial services to prevent and mitigate mental distress and enhance their psychological well-being.

## Figures and Tables

**Table 1:** Gender, age, health disciplines, and questionnaires scores (means (SD)).

Variables	Descriptive (*n* = 915)
Female	753 (82.3%)
Age	22.15 (4.25)
Mental health scales	
Depression (HADS)	5.04 (3.62)
Anxiety (HADS)	9.19 (4.45)
Stress (PSS)	25.59 (9.00)
Psychological well-being (BEP)	82.75 (11.14)
Academic satisfaction (SSS)	23.24 (6.91)

Notes: BEP: Psychological Well-Being Scale; SSS: Scale of Satisfaction with Studies; HADS: Hospital Anxiety and Depression Scale; PSS: Perceived Stress Scale.

**Table 2:** Hierarchical regression of session 1 results.

		Depression (HADS)		Anxiety (HADS)		Stress (PSS)		Psychological Well-Being (BEP)	
		B	SE	B	SE	B	SE	B	SE
Intercept		5.14 ***	0.13	9.62 ***	0.16	40.41 ***	0.32	82.60 ***	0.41
Block 1	Age	0.04	0.03	−0.003	0.03	−0.04	0.07	0.15	0.09
	Gender	−0.61	0.31	−2.44 ***	0.38	−4.61 ***	0.77	0.71	0.96
	*R* ^2^	*0.006*		*0.04*		*0.04*		*0.004*	
Intercept		4.77 ***	0.22	9.53 ***	0.26	40.06 ***	0.53	82.88 ***	0.67
Block 1	Age	0.04	0.03	0.003	0.04	−0.05	0.77	0.13	0.97
	Gender	−0.61	0.31	−2.44 ***	0.38	−4.60 ***	0.07	0.71	0.09
Block 2	1st year	0.63 *	0.28	0.25	0.34	0.42	0.69	−0.72	0.86
	3rd year	0.41	0.32	−0.05	0.39	0.69	0.78	0.12	0.99
	Increase in *R*^2^	*0.006*		*0.001*		*0.001*		*0.001*	
Intercept		4.94 ***	0.26	9.71***	0.32	40.49 ***	0.64	83.16 ***	0.81
Block 1	Age	0.03	0.03	−0.01	0.04	−0.12	0.07	0.16	0.09
	Gender	−0.52	0.31	−2.33 ***	0.38	−4.05 ***	0.76	0.36	0.97
Block 2	1st year	0.68 *	0.28	0.26	0.34	0.31	0.68	−0.65	0.86
	3rd year	0.43	0.32	−0.01	0.39	0.94	0.77	−0.04	0.98
Block 3	Medicine	−0.84 *	0.33	−0.84 *	0.40	−3.66 ***	0.80	2.16 *	1.01
	Pharmaceutical sciences	0.63	0.43	0.21	0.53	0.62	1.05	−2.48	1.33
	Psychology	−0.28	0.29	−0.15	0.36	0.75	0.71	−1.50	0.90
	Increase in *R*^2^	*0.01*		*0.006*		*0.03*		*0.018*	
Intercept		4.77 ***	0.23	9.53 ***	0.29	40.02 ***	0.54	83.63 ***	0.73
Block 1	Age	0.01	0.03	−0.04	0.03	−0.18	0.06	0.22 **	0.08
	Gender	−0.27	0.28	−2.06 ***	0.35	−3.35 ***	0.65	−0.34	0.88
Block 2	1st year	0.42	0.25	−0.02	0.31	−0.41	0.58	0.07	0.78
	3rd year	0.021	0.28	−0.44	0.35	−0.16	0.66	1.08	0.90
Block 3	Medicine	0.36	0.30	0.42	0.38	−0.42	0.70	−1.12	0.95
	Pharmaceutical sciences	0.64	0.38	0.21	0.48	0.63	0.89	−2.49 *	1.21
	Psychology	0.18	0.26	0.33	0.33	1.98 **	0.61	−2.75 **	0.83
Block 4	Academic satisfaction	−0.26 ***	0.02	−0.27 ***	0.02	−0.70 ***	0.04	0.70 ***	0.05
	Increase in *R*^2^	*0.22*		*0.16*		*0.26*		*0.17*	

Note. * *p* < 0.05; ** *p* < 0.01; *** *p* < 0.001; B: Beta coefficients; SE: standard error; HADS: Hospital Anxiety and Depression Scale; PSS: Perceived Stress Scale; BEP: Psychological Well-Being Scale; SSS: Scale of Satisfaction with Studies. Reference categories: women, second year, School of Health Sciences Geneva.

## Data Availability

Data are available upon reasonable request from the corresponding author.
